# Effects of different invitation strategies on participation in a cohort study of Iranian public sector employees: a cluster randomized trial

**DOI:** 10.1186/s12874-021-01405-8

**Published:** 2021-10-09

**Authors:** Rezvan Rajabzadeh, Leila Janani, Seyed Abbas Motevalian

**Affiliations:** 1 Department of Epidemiology, School of Public Health, Iran University of Medical Sciences, Tehran, Iran; 2 Faculty of Medicine, School of Public Health, Imperial Clinical Trials Unit, Imperial College London, London, UK; 3 Research Center for Addiction and Risky Behaviors (ReCARB), Psychosocial Health Research Institute (PHRI), Iran University of Medical Sciences, Tehran, Iran

**Keywords:** Participation rate, Response rate, Invitation, Recruitment, Cohort

## Abstract

**Background:**

Identifying strategies to optimize participation in health studies is one of the major concerns for researchers. The purpose of this study was to evaluate the efficiency of different invitation strategies on participation rate in the Employees’ Health Cohort Study of Iran (EHCSIR).

**Methods:**

Two cluster-randomized trials were carried out to assess the outcomes of different invitation strategies. In the first phase, 7 units with 1880 employees (3 hospitals, 3 health centers, and 1 office) were assigned to the three parallel modes of invitation: 1) invitation letter, 2) phone call and 3) Short Message Service (SMS). In the second phase, 6 hospitals with 1633 employees were allocated to two invitation methods: 1) invitation letter, 2) invitation letter plus EHCSIR project introduction video. All groups were followed up by phone calls. A logistic mixed-effects model was used to compare the effectiveness of the strategies. The cost-effectiveness of the interventions was also compared.

**Results:**

In the first phase, the participation rates in the invitation letter, phone call, and SMS groups were 27.04% (182/673), 21.55% (131/608), and 22.54% (135/599), respectively. Using an invitation letter was significantly more successful than SMS (Adjusted Odds Ratio = 1.80, 95% CI =1.14 to 2.85). Average Cost-Effectiveness Ratios (ACER) were $1.37, $1.42, and $1.55 for the invitation letter, phone call, and SMS, respectively. In the second phase, adding a project introduction video to the invitation letter did not significantly influence the participation rate (Adjusted OR = 0.58, 95% CI =0.24 to 1.36). The ACER was $1.21 for the invitation letter only and $2.01 for the invitation letter plus the introduction video.

**Conclusions:**

In comparison with the phone call and SMS, the invitation letter is the most effective invitation method for public sector employees to participate in a cohort study. Sending an introduction video did not significantly increase the participation rate compared to sending an invitation letter only.

**Supplementary Information:**

The online version contains supplementary material available at 10.1186/s12874-021-01405-8.

## Background

The ability to attract participants is crucial to conduct high-quality research. Failure to achieve the goal of recruiting potential participants can threaten the validity of a study [1, 2]. This failure may lead to methodological, practical, and ethical challenges [3, 4]. Nowadays, recruiting participants has become more difficult, and the response rate in epidemiological studies has decreased dramatically over the past decades [5, 6]. Even in studies that succeed in recruiting a large number of potential participants, the participation level remains low [4, 7]. The participation rates in studies that have a biobank and blood sample collection are less than in other studies [8].

Literature suggests several strategies that can enhance participation rate. For instance, in several studies, paying incentives was shown effective in increasing participation [2, 9, 10], but it imposes more additional financial burden than direct invitation methods [11]. Also, it is recommended that investigators use repeated attempts using different strategies in the invitation to health studies [10, 12]. Because of the variability of the participation rate in epidemiological studies based on the target population and the type of study, a comprehensive investigation of recruitment and maintenance should be one part of the design of any research [1].

## Methods

The objectives of the current study were to explore the impact of different invitation strategies on participation in the EHCSIR project and the cost-effectiveness of those methods.

### Study context

The Employees’ Health Cohort Study of Iran (EHCSIR) is a cohort study of Iranian public sector employees mainly from schools, hospitals and health centers affiliated with the Iran University of Medical Science and the Ministry of Health and Medical Education. The purpose of the study is to identify occupational and non-occupational risk factors for non-communicable diseases, including cardiovascular diseases, cancers, respiratory diseases, vision and hearing disorders, and common psychiatric disorders. All participants take part in several interviews: clinical (past medical history, medications history, smoking), general (migration, ethnicity, lifestyle, and physical activity), dietary habits (including a Food Frequency Questionnaire), job and life stress (job demand/ content, effort-reward imbalance, satisfaction in domains of life), workplace and social health (work performance, quality of life, social capital, workplace social capital), psychological characteristics (personality traits, sleep disorders, smartphone addiction, internet problematic use, aggression, depressive and anxiety disorders). Blood and urine samples are taken for both ad-hoc examinations and storage in a biobank. Blood pressure measurement, electrocardiogram, comprehensive optometric and audiometry assessments, spirometry, anthropometry, and Body Composition Analysis are performed. All of the interviews, clinical and laboratory assessments are performed during 6–7 h on the visit day, and all the deliverable results of investigations are presented to the participants on the same day.

The study population of EHCSIR is more than 15,000 employees working in 43 different work units. Each unit has a liaison officer who is working at the same unit and is nominated by the director of the unit to coordinate arrangements for the participation of the unit’s employees in EHCSIR. Liaison officers are trained in a two-day workshop to learn about the study and the recruitment process. The liaison officers invite colleagues from their own work unit to participate in the study and introduce them to the recruitment unit of EHCSIR project. The recruitment started in July 2017, and the most important challenge of the described recruitment procedure was the low and unmeasurable response rate.

### Study design

We conducted two cluster randomized trials, the first with three and the second with two parallel intervention groups.

### Assignment

In the first phase, 7 clusters with 1880 employees (3 hospitals, 3 health centers, 1 office) were selected. One hospital and one health center were randomly assigned to each invitation group and one administrative unit to the SMS group that had smaller sample size. In the second phase, 6 hospitals with 1633 employees were randomly assigned to two invitation groups.

### Invitation procedure

**Fig. 1:**
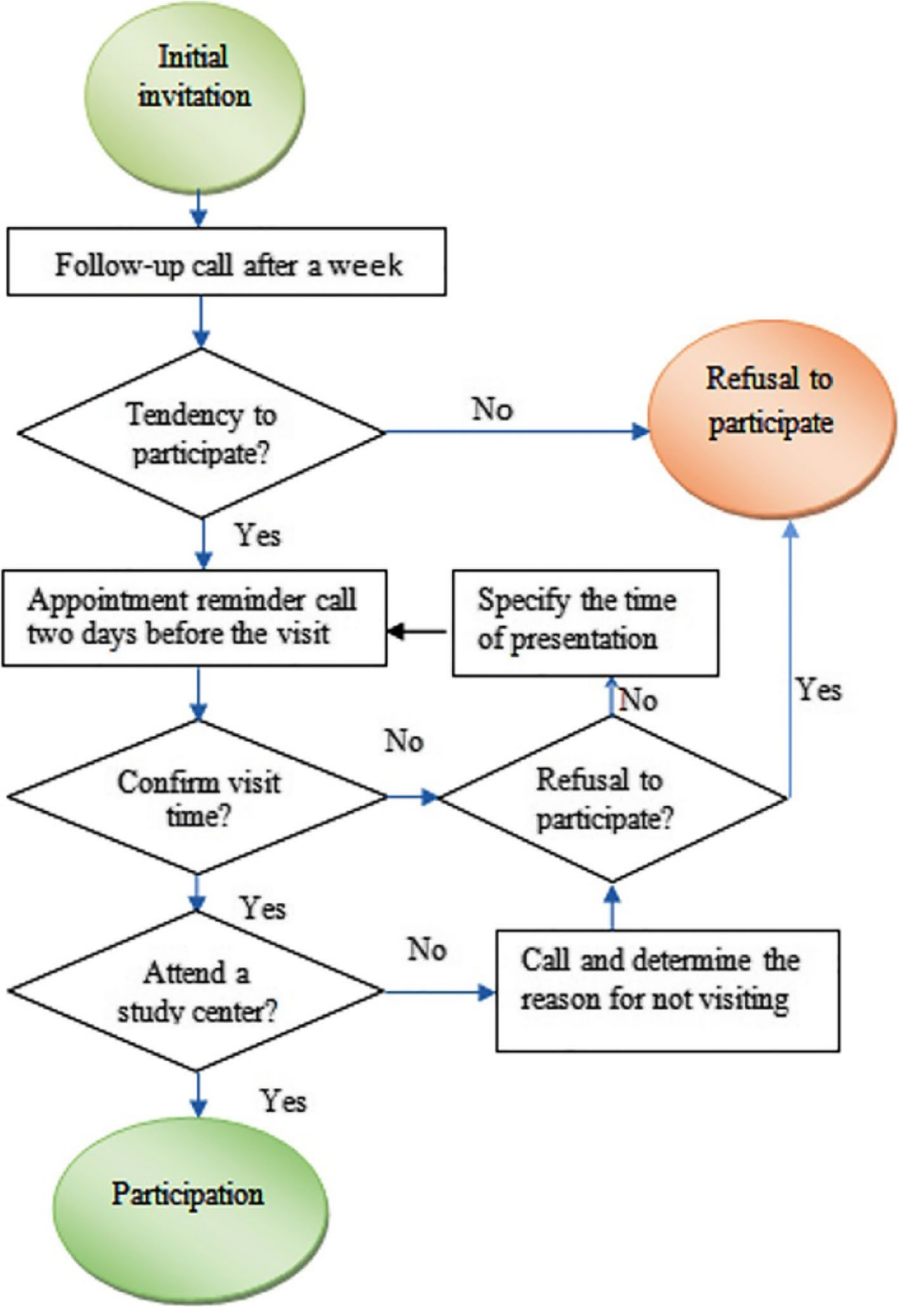
Flowchart of the invitation procedure

As shown in Fig. 1, the invitation procedure consists of several steps. A standard operational protocol was developed for each invitation strategy. A member of the present study made all the contacts with potential participants. In all groups, the content of the provided information about EHCSIR was the same (Additional File 1). Also, liaison officers were trained to follow every unit’s invitation protocol. We have sent the printed invitation letter to the liaison officer and he/she has given them to employees. The demographic and job profile data, besides mobile or home phone numbers were gained from each unit.

The only difference between intervention groups was the mode of the initial invitation.

The first phase interventions consisted of.

1) Invitation letter: the personalized invitation letter that was signed by the EHCSIR’s principal investigator. The letter was printed on EHCSIR letterhead and envelope.

2) Phone call: the employees were invited by phone call.

3) SMS: two identical invitation text messages were sent 48 h apart through the organizational SMS system.

The second phase invitation modes include.

1) Invitation letter: The letter was the most effective strategy based on the first phase results.

2) Invitation letter plus EHCSIR project introduction video: in addition to the letter, the 7-min EHCSIR introduction video was sent to the employees via WhatsApp.

***Follow-up phone call:*** In all invitation strategies, those who did not respond actively to the invitation within 1 week were followed up by a phone call. During this contact, based on the group, the recruiter asked whether they had read the invitation SMS, letter, or watched the video. The recruiter had to make at least three calls.

### Sample size

The sample size was calculated as 600 employees in each group to determine a 20% difference (13% [9] to 33% [13]) in the response rate between the two groups. Intra-cluster correlation coefficient, average cluster size, confidence level of and power were considered 0.02, 200 [14], 95, and 80%, respectively.

### Statistical analysis

Two phases had independent samples and were analyzed independently. Baseline characteristics of employees were summarized by the invitation group. Mean, standard deviation (SD) and median, interquartile range (IQR) were reported for continuous variables, whereas frequencies and proportions were reported for categorical variables. The baseline comparability between the invitation groups was assessed using χ^2^ for categorical variables, in both phases and the Kruskal-Wallis and Mann-Whitney for the continuous variables since their distributions were non-normal in the first phase and the second phase, respectively.

The primary outcome was the participation in EHCSIR as a binary outcome (participation and non-participation) up to a maximum of 3 months after the invitation that was the first contact with the person. The response rate for each intervention group with exact binomial confidence intervals of 95% was calculated. The logistic mixed-effects model with random effects on type of the unit (hospital, health center, and office) and the unit levels to compare the effectiveness of different invitation strategies were applied to estimate the odds ratio of the participation and its 95% confidence interval. The intention to treat analysis approach was used primarily. In a secondary analysis, per-protocol, per-contacted person, analysis approaches were applied.

Intention to treat analysis included all people who were allocated to each invitation group.

The per-contacted analysis included employees that we contacted either in the initial invitation or in the follow-up phone call because we have not called with all of them. The major reason was employees did not answer the phone call. Another reason was the telephone numbers of employees either have been not registered in the database or have been wrong, despite the efforts of the liaisons.

The per-protocol analysis in the invitation letter and SMS groups included contacted employees that had read the letter or SMS before the follow-up call, in the invitation letter plus video group included people who had read the letter and watched the video before the follow-up call, and in the phone group, included all contacted people.

Per-treat analysis, in the first phase, in the invitation letter and SMS groups included contacted employees who had read the letter or SMS before the follow-up call. But people who had not read the letter or SMS were considered phone group. In the phone group, included all contacted people.

Per-treat analysis, in the second phase, in the invitation letter group included contacted employees who had read the letter before the follow-up call. In the invitation letter plus video group, it included people who had read the invitation letter and had watched the video, people who had read the invitation letter but had not watched the video were considered the invitation letter group.

We considered the gender, age, job category, and employment period as individual-level predictors, also the workplace distance from the study center and the Workplace Social Capital (WPSC) as group-level predictors. For the WPSC score, we used the WPSC questionnaire average score of the participants of each center that was completed in EHCSIR. Literature has shown age, gender, job, and distance from the study center were predictors of participation in studies [10, 15–17]. However, we checked the distribution of baseline characteristics between groups. Therefore, we reported our unadjusted and adjusted results for these variables and WPSC. Because of collinearity between the age and the employment duration variables, we just kept the age in the model.

Pairwise comparisons between the invitation letter and phone call have been made using post-estimation commands. All statistical analyses were performed using Stata version 14 and 0.05 was used as the threshold for statistical significance.

### Cost-effectiveness

The total cost of each invitation strategy was determined by summing the cost of all relevant material, including letter paper, envelope, printing, sending the letter, sending SMS, Internet, invitation phone calls, all follow-up calls, and the staff cost. It should be noted that we did not consider the costs of producing the video since the video already had been produced; we used the video before releasing it. Average Cost-Effectiveness Ratio (ACER) was defined as the sum of individual costs divided by the number of participants in EHCSIR.

## Results

**Fig. 2:**
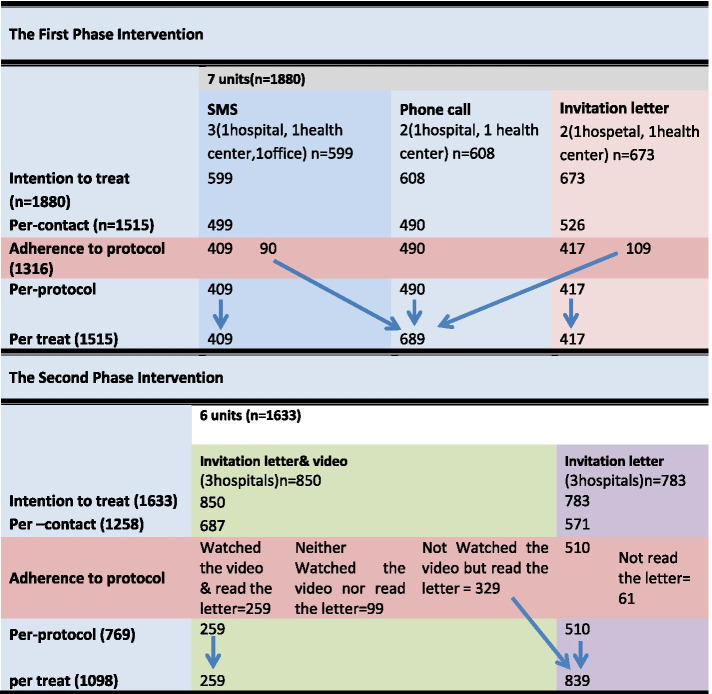
Flow diagram of two phases of study

The flow diagram of two phases was summarized in Fig. 2. Out of 1880 employees invited to the first phase, 1205 (64.10%) were female. The mean and median age of the employees were 42.23 (SD =8.03) and 41(IQR = 36–48) years.

In the second phase, out of 1633 invited employees, 996 (59.15%) were women. Their mean and median age were 42.21 (SD = 7.96) and 41(IQR = 36–48).

Table 1 shows a baseline comparison of the intervention groups. Distributions of baseline characteristics between intervention groups were different significantly (*p* < 0.05). It should be noted that these differences were not important in a medical sense.

**Table 1:** Baseline comparability of the intervention groups

The First Phase (***n*** = 1880)				
Variables		Invitation letter(***n*** = 673)	Phone call (***n*** = 608)	SMS (***n*** = 599)
*Age (year)	**Mean (SD)**	41.33(8.09)	42.43(7.61)	43.04(8.28)
	**Med (IQR)**	39(35–48)	41(37–48)	41(36–50)
**Work Place Social Capital**	**Mean (SD)**	26.98(0.71)	27.34(0.64)	27.71(0.73)
	**Med (IQR)**	27.85 (25.92–27.85)	27.66 (27.66–27.66)	27.09 (27.06–28.66)
*Distance from EHCSIR center (km)	**Minimum-maximum**	7.80–9.50	4.80–43.00	0.50–10.00
	**Med (IQR)**	7.90 (7.90–9.50)	4.80 (4.80–4.80)	7.80 (0.50–7.80)
*Gender **n (%)**	**Male**	198(29.42)	223(36.68)	254(42.40)
	**Female**	475(70.58)	385(63.32)	345(57.60)
*Job category **n (%)**	**Office and clerical worker**	81(12.04)	135(22.20)	197(32.89)
	**Healthcare worker**	473(70.28)	319(52.47)	265(44.24)
	**Service worker**	103(15.30)	116(19.07)	105(17.53)
	**Faculty member**	16(2.38)	38(6.25)	32(5.34)
**The Second Phase (*n*** **= 1633)**				
**Variables**		**Invitation letter (*n*** **= 783)**	**Invitation letter&** **Video (*n*** **= 850)**	
*Age (year)	**mean (SD)**	42.79(7.89)	41.67(7.98)	
	**Med (IQR)**	41(37–48)	40(35–48)	
*Work Place Social Capital	**mean (SD)**	27.90(0.29)	26.61(0.68)	
	**Med (IQR)**	27.66(27.66–28.35)	26.60(25.72–27.41)	
*Distance from EHCSIR center (km)	**Minimum-maximum**	5.5–16.00	9.50–19.00	
	**Med (IQR)**	5.80(5.80–16.00)	18.00(9.50–19.00)	
*Gender **n (%)**	**Male**	341(43.55)	296(34.82)	
	**Female**	442(56.45)	554(65.18)	
*Job category **n (%)**	**Office and clerical worker**	82(10.47)	98(11.53)	
	**Healthcare worker**	512(65.39)	494(58.12)	
	**Service worker**	120(15.33)	213(25.06)	
	**Faculty member**	69(8.81)	45(5.29)	

*statistically significant (***P*** < 0.05)

### Primary analysis

#### Number of calls per respondent

A few numbers attended actively to the invitation within 1 week. In the first phase, we have not accessed the telephone number of 30(1.60%) employees: 1(0.17%), 4(0.66%), and 25(3.71%) in the SMS, phone, and invitation letter group, respectively. The maximum number of follow-up calls was 5 times. Of those who participated, 64.44, 87.63, and 75.27% attended after the first invitation call who were invited by the SMS, the phone call, and the invitation letter, respectively (further details available in Additional file 2: Appendix 1, Table 1).

In the second phase, we have not accessed the telephone number of 98(6.00%) employees: 9(1.06%) and 89(11.37%) in the invitation letter plus video and the invitation letter group, respectively. The maximum number of follow-up calls was 5 times. Of those who participated, 92.42 and 89.08% participated after the first invitation call who were invited by the invitation letter alone and the invitation letter plus video, respectively (further details available in Additional file 2: Appendix 1, Table 2).

#### Intention to treat analysis

The overall participation rate in the current study was 25.22%. The effectiveness of the interventions on the participation rates are given in Table **2**. The invitation letter was the highest response rate (27.04%) compared to the phone call and SMS strategies. Also, there was a small difference between the participation rate on the phone call and SMS methods (21.55% vs. 22.54).

**Table 2:** Effectiveness of interventions in participation to EHCSIR (Using mixed-effects logistic regression models)

The First Phase (***n*** = 1880)							
Analysis approach	Intervention	n	Participation % (95% CI)	OR _**unadjusted**_ (95% CI)	***P***-value	OR _**adjusted**_^a *^ (95% CI)	***P***-value
**Intention to Treat (n = 1880)**	**SMS**	135/599	22.54 (19.25–26.10)	1		1	
	**Phone call**	131/608	21.55 (18.34–25.03)	1.30 (0.47–3.64)	0.612	0.77 (0.21–2.78)	0.692
	**Invitation letter**	182/673	27.04 (23.72–30.57)	1.88 (1.20–2.95)	0.006	1.80 (1.14–2.85)	0.012
**Contacted person (*n*** **= 1515)**	**SMS**	135/499	27.05 (23.20–31.18)	1		1	
	**Phone call**	131/490	26.73 (22.86–30.89)	1.20 (0.47–3.07)	0.699	0.79 (0.25–2.49)	0.682
	**Invitation letter**	182/526	34.60 (30.54–38.84)	1.78 (1.13–2.80)	0.013	1.67 (1.05–2.66)	0.032
**Per –protocol (*n*** **= 1316)**	**SMS**	122/409	29.83 (25.43–34.52)	1		1	
	**Phone call**	131/490	26.73 (22.87–30.89)	1.02 (0.41–2.54)	0.966	0.65 (0.22–1.92)	0.434
	**Invitation letter**	166/417	39.81 (35.07–44.69)	1.77 (1.09–2.88)	0.022	1.60 (0.98–2.64)	0.063
**Per-treat (n = 1515)**	**SMS**	122/409	29.83 (25.43–34.52)	1		1	
	**Phone call**	160/689	23.22 (20.12–26.56)	0.52 (0.32–0.86)	0.009	0.45 (0.27–0.73)	0.001
	**Invitation letter**	166/417	39.81 (35.08–44.69)	1.80 (1.14–2.85)	0.012	1.59 (1.00–2.53)	0.048
**The Second Phase (n = 1633)**							
**Analysis approach**	**Intervention**	**n**	**Participation % (95% CI)**	**OR** ^**b**^_**unadjusted**_ **(95% CI)**	***P*-value**	**OR** ^**b**^_**adjusted**_ **(95% CI)**	***P*-value**
**Intention to Treat (n = 1633)**	**Invitation letter**	264/783	33.72 (30.41–37.15)	1		1	
	**Invitation letter& video**	174/850	20.47 (17.81–23.34)	0.70 (0.28–1.71)	0.431	0.58 (0.24–1.36)	0.209
**Contacted person (*n*** **= 1258)**	**Invitation letter**	264/571	46.23 (42.08–50.42)	1		1	
	**Invitation letter& video**	174/687	25.33 (22.11–28.75)	0.55 (0.24–1.25)	0.151	0.41 (0.15–1.07)	0.069
**Per –protocol (*n*** **= 768)**	**Invitation letter**	261/510	51.18 (46.74–55.60)	1		1	
	**Invitation letter& video**	78/259	30.12 (24.59–36.10)	0.60 (0.19–1.95)	0.398	0.27 (0.08–0.85)	0.026
**Per-treat (*n*** **= 1098)**	**Invitation letter**	356/839	42.43 (39.06–45.86)	1		1	
	**Invitation letter& video**	78/259	30.12 (24.59–36.10)	1.03 (0.71–1.47)	0.889	1.27 (0.87–1.85)	0.209

^a^ Random effect: type of center and center

^b^ Random effect: center

*Adjusted by the gender, age, job category, work place distance from EHCSIR center, and work place social capital

The participation rate by the invitation letter alone and the invitation letter plus the EHCSIR project introduction video were 33.72 and 20.47%, respectively.

The results of the logistic mixed-effects models showed that in the first phase, the invitation letter compared to SMS significantly increased participation (Adjusted OR = 1.80, 95% CI = 1.14–2.85). The comparison between the invitation letter versus phone call showed no significant difference between their effect on participation (Unadjusted OR = 1.44, 95%CI = 0.52–3.97), (Adjusted OR = 2.33, 95%CI = 0.64–8.49) (further details available in Additional file 2: Appendix 2).

In the second phase, the participation rate of those who were sent the EHCSIR project introduction video together with the invitation letter was not significantly different from the invitation letter-only group (Adjusted OR = 0.58, 95% CI = 0.24–1.36).

### Secondary analyses

#### Per-contacted analysis

The analysis based on the contacted people showed that the invitation letter was significantly better than the SMS (Adjusted OR = 1.67, 95% CI = 1.05–2.66) (see Table **2**). The comparison between the invitation letter and phone call showed no significant difference between the effect of invitation letter and phone call strategies on participation (Unadjusted OR = 1.48, 95%CI = 0.59–3.72), (Adjusted OR = 2.12, 95%CI = 0.66–6.82).

In the second phase, adding video to the invitation letter could not significantly increase the response versus only the invitation letter (Adjusted OR = 0.41, 95% CI = 0.15–1.07) (see Table **2**).

#### Per-protocol analysis

In the first phase, among contacted people in the SMS and the invitation letter groups, 409 and 417 people mentioned that they had read the SMS and the invitation letter before the follow-up contact, respectively. We considered people who read the SMS or the invitation letter, also all contacted people (490) in the phone group, as the people who had followed the invitation protocol.

The analysis based on the adherence to invitation protocol showed that the phone call (Adjusted OR = 0.65, 95% CI = 0.22–1.92) and invitation letter (Adjusted OR = 1.60, 95% CI = 0.98–2.64) did not significantly affect the participation compared with the SMS method (see Table 2). The comparison between the invitation letter and phone call showed that there was no significant difference between them (Unadjusted OR = 1.74, 95%CI = 0.71–4.26), (Adjusted OR = 2.48, 95%CI = 0.82–7.47). The Workplace distance from EHCSIR center and WPSC did not affect the participation rate significantly (*P* > 0.05).

In the second phase, among contacted people in the invitation letter plus video group, 259 people had read the letter and watched the video before the follow-up contact. Also, in the invitation letter group, 510 people had read the invitation letter before the follow-up contact. We considered them as the person who has followed the invitation protocol.

The analysis based on the adherence to invitation protocol showed that adding the study introduction video to the invitation letter significantly reduced the response versus the invitation letter only (Adjusted OR = 0.27, 95% CI = 0.08–0.85) (see Table 2).

#### Per-treat analysis

In the first phase, among contacted people in the letter (526) and SMS (499) groups, people who had not read the letter or SMS were considered the phone group. The per-treat analysis showed that participation in the invitation letter group was significantly higher than the SMS group (Adjusted OR = 1.59, 95% CI = 1.00–2.53), but participation in the phone call group was significantly lower than SMS group (Adjusted OR = 0.45, 95% CI = 0.27–0.73) (see Table 2). The comparison between the invitation letter and phone call showed that using the invitation letter increased participation in the study significantly compared with the phone call strategy (Unadjusted OR = 3.47, 95%CI = 2.19–5.48), (Adjusted OR = 3.55, 95%CI = 2.24–5.64).

In the second phase, among 850 whom people were invited by the invitation letter plus video, only 259 (30.47%) read the letter and watched the video, 329 (47.89%) just read the letter; as a result, in per-treat analysis, these people were considered the invitation letter plus video and the invitation letter groups, respectively. In the invitation letter group, 510 (71.97%) read the invitation letter. Finally, 839 and 259 people were considered as the invitation letter and the invitation letter plus video group, respectively.

The per-treat analysis showed that adding video to the invitation letter did not significantly influence the response versus only the invitation letter (Adjusted OR = 1.27, 95% CI = 0.87–1.85) (see Table 2).

### Cost-effectiveness

Table 3 shows cost-effectiveness analysis. In the first phase, the average cost per participation was $1.37, $1.42, and $1.55 for the invitation letter, phone call, and SMS groups, respectively.

**Table 3:** Cost-effectiveness of invitation strategies

	Invitation	Participation rate^**a**^	Cost per invitation	ACER^**b**^
**The First Phase**	**SMS** staff time, sending SMS, phone calls	22.54	$0.35	$1.55
	**Phone call** staff time, phone call	21.55	$0.31	$1.42
	**Invitation letter** staff time, letter paper, envelope, printing, sending the letter, phone calls	27.04	$0.37	$1.37
**The Second Phase**	**Invitation letter** staff time, letter paper, envelope, printing, sending the letter, phone calls	33.72	$0.41	$1.22
	**Invitation letter plus video** staff time, letter paper, envelope, printing, sending the letter, phone calls**,** internet	20.47	$0.41	$2.01

^a^ Intention to treat

^b^Average Cost Effectiveness Ratio (ACER) = cost (c) /Effectiveness (E)

In the second phase, the average cost per participation was $1.21 and $2.01 for the invitation letter, and the invitation letter plus video groups, respectively.

## Discussion

This study assessed the impact of several invitation strategies to participate in an employees’ cohort study. We achieved the highest response rate using the invitation letter. The invitation letter successfully improved participation compared with SMS. However, the phone call did not significantly influence participation compared with the SMS method. The invitation letter had the lowest average cost per participation in the study. Sending the study introduction video did not significantly affect participation compared with sending only the invitation letter. Those who watched the video were more likely to participate than those who did not. Perhaps it is because those who are intrinsically interested in participating in the study are more likely to watch a video about the study.

Based on the social exchange theories, prior notification mailings help build confidence in a study [10]. Besides, behavior theory suggests that the personalized letter could strengthen the study’s relationship with participants and, as a result, increases the participation rate [10]. In several large studies, mass mailing was a critical invitation strategy [7, 13, 18].

In our study, the participation rates in phases I and II between the two invitation letter groups were different. It could be because our target population was employees of one university who could share their experiences and information during progressing of the EHCSIR project.

We could not compare our results with previous studies accurately because evidence about the effectiveness and cost-effectiveness of invitation strategies in cohort studies is limited [2, 11]. Moreover, the participation rate and the reasons that people take part in health studies are variable according to the characteristics of the target population [3, 19]. However, consistent with our study, some studies have suggested that invitation letter was more cost-effective [20]. In another study, invitation by two letters was more effective and more cost-effective than invitations by one letter. Also, they reported the highest efficiency for one letter plus phone call in people whose phone number was available [9].

In our study, it was not possible to assess the phone reminder effect because we used phone call follow-up as one of our invitation steps in all our intervention groups. The previous studies [1, 6, 9, 15] indicated that reminder and follow-up, especially phone call reminders, were effective in increasing the response rate. Though, in another study, sending a reminder letter did not have a significant impact on the response rate [13].

Another study showed that there is no difference between sending a letter and an e-mail, but the cost of sending a letter was higher [6]. The cost per participation in all invitation strategies in our study was lower in all invitation strategies in our study than in other research [6, 10, 21].

Age, job category, and distance from the study center were the determinants of the participation rate. By controlling other factors, participation has also increased with age. In most studies, despite the differences in the target population, the relationship between the age and participation rate has been shown, and it has been concluded that the likelihood of participation of older people has been more than younger people [15, 16]. In this study, the women’s participation rate was slightly higher than men, but no significant association was observed. This result is consistent with a study by Banks et al. [17], but in other studies, the participation of women has been significantly higher than men [10, 16].

The strengths of this study are its methodology, sufficient sample size, and availability of demographic information of employees without any missing data. Besides, the cost-effectiveness of the interventions has been calculated.

The main limitation of this study was the low number of clusters; in this regard, the balance between intervention groups did not meet. Nevertheless, we had controlled known and measured predictors of participation; other kinds of predictors have not been controlled obviously. As a result, our results suffer from somewhat uncertainty.

## Conclusions

Compared to the phone call and SMS, the invitation letter is the most successful method of inviting public sector employees to participate in a cohort study. Also, the invitation letter was the most cost-effective strategy in comparison with the phone call and SMS. There is a no significant impact on sending the study introduction video in increasing the likelihood of participation. To make sure about the video’s effect on the response rate, it seems that we should be looking for a strategy that increases the likelihood of watching the video.

## Supplementary Information

**Additional file 1:.**

**Additional file 2:.**

## Data Availability

The datasets generated and analyzed during the current study are not publicly available because of containing information that could potentially identify study participants. However, de-identified data are available from the corresponding author on reasonable request.

## Abbreviations

EHCSIR: Employees’ Health Cohort Study of Iran
SMS: Short Message Service
OR: Odds Ratio
CI: Confidence Interval
ACER: Average Cost-Effectiveness Ratio
ICER: Incremental Cost-Effectiveness Ratio
WPSC: Workplace Social Capital
ITT: Intention to Treat
SD: Standard Deviation
Med (IQR): Median (Inter Quartile Range)
n: Number
