# Supplementary material for: Effects of different invitation strategies on participation in a cohort study of Iranian public sector employees: a cluster randomized trial
Source: BMC Med Res Methodol. 2021 Oct 9;21:206. doi: 10.1186/s12874-021-01405-8 (PMC8502307; doi:10.1186/s12874-021-01405-8)
Supplement: Supplementary file 1 — Additional file 1:. [file 12874_2021_1405_MOESM1_ESM.docx]

The following sections contain Appendices to the study: "**Effects of different invitation strategies on participation in a cohort study of Iranian public sector employees: a cluster randomized trial"**

Rezvan Rajabzadeh ^1^, Leila Janani ^2^, Seyed Abbas Motevalian ^1, 3*^

^1^Department of Epidemiology, School of Public Health, Iran University of Medical Sciences, Tehran, Iran

^2^ Faculty of Medicine, School of Public Health, Imperial Clinical Trials Unit, Imperial College London

^3^Research Center for Addiction and Risky Behaviors (ReCARB), Psychosocial Health Research Institute (PHRI), Iran University of Medical Sciences, Tehran, Iran

Rezvan Rajabzadeh. rajabzade.61@gmail.com. ORCID ID: 0000-0001-6567-0724

Leila Janani. l.janani@imperial.ac.uk. ORCID ID: 0000-0002-3370-9310

*Corresponding authors: Seyed Abbas Motevalian. Department of Epidemiology, School of Public Health, Iran University of Medical Sciences. Research Center for Addiction and Risky Behaviors (ReCARB), Psychosocial Health Research Institute (PHRI), Iran University of Medical Sciences Tel: +982186702503. E-mail: motevalian.a@iums.ac.ir. ORCID ID: 0000-0002-0404-4495

Supplementary 1 The content of the invitation

Date:

Number:

**In the name of God**

**Dear**

**Subject:** Invitation to participate in the Employees' Health Cohort Study of Iran (EHCSIR)

We declare that the Employees' Health Cohort Study of Iran (EHCSIR) is in process at Iran University of Medical Sciences. All the employees (Permanent, contract, and contractual) of Iran University of Medical Sciences are eligible to participate in this study. In this study, comprehensive assessments of your health status, such as blood and urine tests, electrocardiogram, optometry, and audiometry will be performed and the results will be provided to you. Individuals' information will be recorded secretly and will be used only in medical researches. Evaluations of this study are conducted annually by telephone and every four years in person. Participation in this study is completely voluntary and the day of the visit will be considered as an administrative assignment for the participants. Now your turn to participate in this study has arrived and you have time until next week, when you will be telephoned, to decide about your tendency to participate in the study and determine your desired date from Saturday up to Wednesday on …….

You can get more information from the coordinator of your unit, Ms. /Mr. …, or by contacting EHCSIR's Study Center (Tel :....).

**"I care about my health and that of future generations. I participate in the EHCSIR."**

**Accept my endless gratitude**

**Dr. Seyed Abbas Motevalian**

**The Principal Investigator of the Employees' Health Cohort Study of Iran**
