# Supplementary material for: Effects of different invitation strategies on participation in a cohort study of Iranian public sector employees: a cluster randomized trial
Source: BMC Med Res Methodol. 2021 Oct 9;21:206. doi: 10.1186/s12874-021-01405-8 (PMC8502307; doi:10.1186/s12874-021-01405-8)
Supplement: Supplementary file 2 — Additional file 2:. [file 12874_2021_1405_MOESM2_ESM.docx]

The following sections contain Appendices to the study: **"Effects of different invitation strategies on participation in a cohort study of Iranian public sector employees: a cluster randomized trial"**

Rezvan Rajabzadeh ^1^, Leila Janani ^2^, Seyed Abbas Motevalian ^1, 3*^

^1^Department of Epidemiology, School of Public Health, Iran University of Medical Sciences, Tehran, Iran

^2^ Faculty of Medicine, School of Public Health, Imperial Clinical Trials Unit, Imperial College London

^3^Research Center for Addiction and Risky Behaviors (ReCARB), Psychosocial Health Research Institute (PHRI), Iran University of Medical Sciences, Tehran, Iran

Rezvan Rajabzadeh. rajabzade.61@gmail.com. ORCID ID: 0000-0001-6567-0724

Leila Janani. l.janani@imperial.ac.uk. ORCID ID: 0000-0002-3370-9310

*Corresponding authors: Seyed Abbas Motevalian. Department of Epidemiology, School of Public Health, Iran University of Medical Sciences. Research Center for Addiction and Risky Behaviors (ReCARB), Psychosocial Health Research Institute (PHRI), Iran University of Medical Sciences Tel: +982186702503. E-mail: motevalian.a@iums.ac.ir. ORCID ID: 0000-0002-0404-4495

**Appendix1**

**Table 1 Number of calls based on intervention group and outcome in phase1**

| **Call** | **Group** |  | **Number of call** | | | | | | **Total n(%)** |
| --- | --- | --- | --- | --- | --- | --- | --- | --- | --- |
|  |  |  | **0 n(%)** | **1 n(%)** | **2 n(%)** | **3 n(%)** | **4 n(%)** | **5 n(%)** |  |
| **Total call** | **SMS** | **NP** | 3(0.65) | 161(34.70) | 139(29.26) | 83(17.89) | 62(13.36) | 16(3.45) | 464(100) |
|  |  | **P** | 2 (1.48) | 57(42.22) | 44(32.59) | 15(11.11) | 13(9.63) | 4(2.96) | 135(100) |
|  |  | **T** | 5(0.83) | 218(36.39) | 183(30.55) | 98(16.36) | 75(12.52) | 20(3.34) | 599(100) |
|  | **Phone** | **NP** | 15(3.14) | 185(38.78) | 135(28.30) | 123(25.79) | 16.3.35) | 3(0.63) | 477(100) |
|  |  | **P** | 0 | 74(56.49) | 23(17.56) | 27(20.61) | 5(3.82) | 2(1.53) | 131(100) |
|  |  | **T** | 26(4.28) | 248(40.79) | 158(25.99) | 150(24.67) | 21(3.45) | 5(0.82) | 608(100) |
|  | **Invitation letter** | **NP** | 20(4.07) | 253(51.53) | 101(20.57) | 87(17.72) | 25(5.09) | 5(1.02) | 491(100) |
|  |  | **P** | 8 (4.40) | 75(41.21) | 64(35.16) | 27(14.84) | 5(2.75) | 3(1.65) | 182(100) |
|  |  | **T** | 28(4.16) | 328(48.74) | 165(24.52) | 114(16.94) | 30(4.46) | 8(1.19) | 673(100) |
| **Successful call** | **SMS** | **NP** | 95(20.47) | 242(52.16) | 97(20.91) | 28(6.03) | 2(0.43) | 0 | 464(100) |
|  |  | **P** | 9 (6.67) | 78(57.78) | 39(28.89) | 8(5.93) | 0 | 1(0.74) | 135(100) |
|  |  | **T** | 104(17.36) | 320(53.42) | 136(22.70) | 36(6.01) | 2(0.33) | 1(0.17) | 599(100) |
|  | **Phone** | **NP** | 133(27.88) | 277(58.07) | 53(11.11) | 14(2.94) | 0 | - | 477(100) |
|  |  | **P** | - | 103(78.63) | 24(18.32) | 3(2.29) | 1(0.76) | - | 131(100) |
|  |  | **T** | 133(25.49) | 380(62.50) | 77(12.66) | 17(2.80) | 1(0.16) | - | 608(100) |
|  | **Invitation letter** | **NP** | 144(29.33) | 288(58.66) | 48(9.78) | 9(1.83) | 2(0.41) | - | 491(100) |
|  |  | **P** | 19 (10.44) | 118(64.84) | 44(24.18) | 1(0.55) | 0 | - | 182(100) |
|  |  | **T** | 163(24.22) | 406(60.33) | 92(13.67) | 10(1.49) | 2(0.30) | - | 673(100) |
| **Unsuccessful call** | SMS | **NP** | 215(46.34) | 111(23.92) | 55(11.85) | 52(11.21) | 29(6.25) | 2(0.43) | 646(100) |
|  |  | **P** | 87(64.44) | 30(22.22) | 10(7.41) | 5(3.70) | 3(2.22) | 0 | 135(100) |
|  |  | **T** | 302(50.42) | 141(23.54) | 65(10.85) | 57(9.52) | 32(5.34) | 2(0.33) | 599(100) |
|  | Phone | **NP** | 221(46.33) | 115(24.11) | 65(13.63) | 69(14.47) | 7(1.47) | - | 477(100) |
|  |  | **P** | 90(65.65) | 20(15.27) | 16(12.21) | 5(3.82) | 0 | - | 131(100) |
|  |  | **T** | 311(51.15) | 135(22.20) | 81(13.32) | 74(12.17) | 7(1.15) | - | 608(100) |
|  | Invitation letter | **NP** | 253(51.53) | 126(25.66) | 50(10.18) | 48(9.57) | 14(2.85) | 1(0.20) | 491(100) |
|  |  | **P** | 110(60.4) | 47(25.82) | 17(9.34) | 4(1.65) | 5(2.75) | 0 | 182(100) |
|  |  | **T** | 363(53.94) | 173(25.71) | 67(9.96) | 52(7.43) | 19(2.82) | 1(0.15) | 673(100) |
| NP: Non Participants  P: participants  T: Total | | | | | | | | | |

**Table 2 Number of calls based on intervention group and outcome in phase2**

| **Call** | **Group** |  | **Number of call** | | | | | | **Total n(%)** |
| --- | --- | --- | --- | --- | --- | --- | --- | --- | --- |
|  |  |  | **0 n(%)** | **1 n(%)** | **2 n(%)** | **3 n(%)** | **4 n(%)** | **5 n(%)** |  |
| **Total call** | **Invitation letter** | **NP** | 81(15.61) | 203(39.11) | 134(25.82) | 87(16.76) | 13(2.50) | 1(0.19) | 519(100) |
|  |  | **P** | 1 (0.38) | 140(53.03) | 78(29.55) | 36(13.64) | 8(3.03) | 1(0.38) | 264(100) |
|  |  | **T** | 82(10.47) | 343(43.81) | 212(27.08) | 123(15.71) | 21(2.68) | 2(0.26) | 783(100) |
|  | **Invitation letter & video** | **NP** | 1(0.15) | 193(28.55) | 226(33.43) | 163(24.11) | 81(11.98) | 12(1.78) | 676(100) |
|  |  | **P** | 4 (2.30) | 72(41.38) | 64(36.78) | 25(14.37) | 7(4.02) | 2(1.15) | 174(100) |
|  |  | **T** | 5(0.59) | 265(31.18) | 290(34.12) | 188(22.12) | 88(10.35) | 14(1.65) | 850(100) |
| **Successful call** | **Invitation letter** | **NP** | 201(38.73) | 264(50.87) | 48(9.25) | 6(1.16) | - | - | 519(100) |
|  |  | **P** | 74 (28.03) | 170(64.39) | 20(7.58) | 0 | - | - | 264(100) |
|  |  | **T** | 275(35.12) | 434(55.43) | 68(8.68) | 6(0.77) | - | - | 783(100) |
|  | **Invitation letter & video** | **NP** | 161(23.82) | 354(52.37) | 161(23.82) | 0 | - | - | 676(100) |
|  |  | **P** | 33 (18.97) | 122(70.11) | 16(9.20) | 3(1.72) | - | - | 174(100) |
|  |  | **T** | 194(22.82) | 476(56.00) | 177(20.82) | 3(0.35) | - | - | 850(100) |
| **Unsuccessful call** | **Invitation letter** | **NP** | 276(53.18) | 114(21.97) | 76(14.64) | 48(9.25) | 5(0.96) | - | 519(100) |
|  |  | **P** | 122(46.21) | 84(31.82) | 31(11.74) | 22(8.33) | 5(1.89) | - | 264(100) |
|  |  | **T** | 398(50.83) | 198(25.29) | 107(13.67) | 70(8.94) | 10(1.28) | - | 783(100) |
|  | **Invitation letter & video** | **NP** | 286(42.31) | 151(22.34) | 67(9.91) | 123(18.20) | 49(7.25) | - | 676(100) |
|  |  | **P** | 73(41.95) | 64(36.78) | 27(15.52) | 8(4.60) | 2(1.15) | - | 174(100) |
|  |  | **T** | 359(42.24) | 215(25.29) | 94(11.06) | 131(15.41) | 51(6.00) | - | 850(100) |
| NP: Non Participants  P: participants  T: Total | | | | | | | | | |

**Appendix2**

Table 3 Effectiveness of interventions in participation to EHCSIR (Using mixed effects logistic regression models)

| **The First Phase ^a^** | | | | | | | | |
| --- | --- | --- | --- | --- | --- | --- | --- | --- |
|  | | | OR^**^ | P-value | | | 95% CI | |
| **Intention to Treat(n=1880)** | | | | | | | | |
| **Intervention** | **SMS** | | 1 | |  | | | |
|  | **Phone call** | | 0.77 | | 0.692 | 0.21 | | 2.78 |
|  | **Invitation letter** | | 1.80 | | 0.012 | 1.14 | | 2.85 |
| **Gender** | **Male** | | 1 | |  | | | |
|  | **Female** | | 1.22 | | 0.146 | 0.93 | | 1.60 |
| **Age** |  | | 1.03 | | <0.001 | 1.02 | | 1.05 |
| **Job category** | **Office and clerical worker** | | 1 | |  | | | |
|  | **Health care worker** | | 1.59 | | 0.006 | 1.14 | | 2.22 |
|  | **Service worker** | | 2.42 | | <0.001 | 1.63 | | 3.60 |
|  | **Faculty member** | | 0.43 | | 0.059 | 0.18 | | 1.03 |
| **Workplace Distance from EHCSIR center** | | | 1.04 | | 0.168 | 0.98 | | 1.10 |
| **Workplace Social Capital** | | | 1.37 | | 0.384 | 0.67 | | 2.78 |
| **Contacted person (n=1515)** | | | | | | | | |
| **Intervention** | | **SMS** | 1 | |  | | | |
|  |  | **Phone call** | 0.79 | | 0.682 | 0.25 | | 2.49 |
|  |  | **Invitation letter** | 1.67 | | 0.032 | 1.05 | | 2.66 |
| **Gender** | | **Male** | 1 | |  | | | |
|  |  | **Female** | 1.34 | | 0.045 | 1.01 | | 1.77 |
| **Age** | | | 1.03 | | <0.001 | 1.01 | | 1.05 |
| **Job category** | | **Office and clerical worker** | 1 | |  | | | |
|  |  | **Health care worker** | 1.54 | | 0.013 | 1.10 | | 2.17 |
|  |  | **Service worker** | 2.71 | | 0.000 | 1.78644 | | 4.11 |
|  |  | **Faculty member** | 0.45 | | 0.078 | 0.19 | | 1.09 |
| **Workplace Distance from EHCSIR center** | | | 1.03 | | 0.210 | 0.98 | | 1.09 |
| **Workplace Social Capital** | | | 1.28 | | 0.446 | 0.68 | | 2.44 |
| **Per –protocol (n=1316)** | | | | | | | | |
| **Intervention** | | **SMS** | 1 | |  | | | |
|  |  | **Phone call** | 0.65 | | 0.434 | 0.22 | | 1.92 |
|  |  | **Invitation letter** | 1.60 | | 0.063 | 0.98 | | 2.64 |
| **Gender** | | **Male** | 1 | |  | | | |
|  |  | **Female** | 1.36 | | 0.046 | 1.01 | | 1.83 |
| **Age** | | | 1.03 | | 0.001 | 1.01 | | 1.05 |
| **Job category** | | **Office and clerical worker** | 1 | |  | | | |
|  |  | **Health care worker** | 1.52 | | 0.018 | 1.08 | | 2.18 |
|  |  | **Service worker** | 3.08 | | 0.000 | 1.99 | | 4.77 |
|  |  | **Faculty member** | 0.57 | | 0.222 | 0.23 | | 1.40 |
| **Work Place Distance from EHCSIR center** | | | 1.03 | | 0.188 | 0.98 | | 1.09 |
| **Work Place Social Capital** | | | 1.25 | | 0.470 | 0.68 | | 2.31 |
| **Per-treat (n=1515)** | | | | | | | | |
| **Intervention** | | **SMS** | 1 | |  | | | |
|  |  | **Phone call** | 0.45 | | 0.001 | 0.27 | | 0.73 |
|  |  | **Invitation letter** | 1.59 | | 0.048 | 1.00 | | 2.53 |
| **Gender** | | **Male** | 1 | |  | | | |
|  |  | **Female** | 1.32 | | 0.059 | 0.99 | | 1.75 |
| **Age** | | | 1.03 | | <0.001 | 1.01 | | 1.05 |
| **Job category** | | **Office and clerical worker** | 1 | |  | | | |
|  |  | **Health care worker** | 1.56 | | 0.011 | 1.11 | | 2.20 |
|  |  | **Service worker** | 2.82 | | <0.001 | 1.86 | | 4.29 |
|  |  | **Faculty member** | 0.50 | | 0.125 | 0.21 | | 1.21 |
| **Workplace Distance from EHCSIR center** | | | 1.04 | | 0.068 | 1.00 | | 1.09 |
| **Workplace Social Capital** | | | 1.26 | | 0.467 | 0.68 | | 2.33 |
| **The Second Phase ^b^** | | | | | | | | |
|  | | | OR^**^ | | P-value | | 95% CI | |
| **Intention to Treat(n=1633)** | | | | | | | | |
| **Intervention** | **Invitation letter** | | 1 | |  | | | |
|  | **Invitation letter& video** | | 0.58 | | 0.209 | | 0.24 | 1.36 |
| **Gender** | **Male** | | 1 | |  | | | |
|  | **Female** | | 0.86 | | 0.286 | | 0.65 | 1.14 |
| **Age** |  | | 1.03 | | <0.001 | | 1.02 | 1.05 |
| **Job category** | **Office and clerical worker** | | 1 | |  | | | |
|  | **Health care worker** | | 0.86 | | 0.525 | | 0.61 | 1.29 |
|  | **Service worker** | | 1.11 | | 0.659 | | 0.70 | 1.74 |
|  | **Faculty member** | | 0.09 | | <0.001 | | 0.04 | 0.20 |
| **Workplace Distance from EHCSIR center** | | | 0.89 | | <0.001 | | 0.84 | 0.94 |
| **Workplace Social Capital** | | | 0.53 | | 0.015 | | 0.33 | 0.89 |
| **Contacted person (n=1258)** | | | | | | | | |
| **Intervention** | **Invitation letter** | | 1 | |  | | | |
|  | **Invitation letter& video** | | 0.41 | | 0.069 | | 0.15 | 1.07 |
| **Gender** | **Male** | | 1 | |  | | | |
|  | **Female** | | 0.83 | | 0.215 | | 0.61 | 1.12 |
| **Age** | | | 1.04 | | <0.001 | | 1.02 | 1.06 |
| **Job category** | **Office and clerical worker** | | 1 | |  | | | |
|  | **Health care worker** | | 0.88 | | 0.521 | | 0.59 | 1.31 |
|  | **Service worker** | | 1.34 | | 0.238 | | 0.82 | 2.17 |
|  | **Faculty member** | | 0.11 | | <0.001 | | 0.05 | 0.26 |
| **Workplace Distance from EHCSIR center** | | | 0.92 | | 0.013 | | 0.86 | 0.98 |
| **Workplace Social Capital** | | | 0.56 | | 0.048 | | 0.32 | 0.99 |
| **Per –protocol (n=768)** | | | | | | | | |
| **Intervention** | **Invitation letter** | | 1 | |  | | | |
|  | **Invitation letter& video** | | 0.27 | | 0.026 | 0.08 | | 0.85 |
| **Gender** | **Male** | | 1 | |  | | | |
|  | **Female** | | 0.92 | | 0.671 | 0.63 | | 1.34 |
| **Age** | | | 1.04 | | 0.002 | 1.01 | | 1.06 |
| **Job category** | **Office and clerical worker** | | 1 | |  | | | |
|  | **Health care worker** | | 0.73 | | 0.200 | 0.45 | | 1.18 |
|  | **Service worker** | | 1.11 | | 0.746 | 0.59 | | 2.11 |
|  | **Faculty member** | | 0.11 | | <0.001 | 0.04 | | 0.34 |
| **Workplace Distance from EHCSIR center** | | | 0.91 | | 0.013 | 0.84 | | 0.98 |
| **Workplace Social Capital** | | | 0.35 | | 0.002 | 0.18 | | 0.68 |
| **Per-treat (n=1098)** | | | | | | | | |
| **Intervention** | **Invitation letter** | | 1 |  | | | | |
|  | **Invitation letter& video** | | 1.27 | 0.209 | | | 0.87 | 1.85 |
| **Gender** | **Male** | | 1 |  | | | | |
|  | **Female** | | 0.75 | | 0.082 | 0.55 | | 1.04 |
| **Age** | | | 1.04 | | <0.001 | 1.02 | | 1.06 |
| **Job category** | **Office and clerical worker** | | 1 | |  | | | |
|  | **Health care worker** | | 0.87 | | 0.512 | 0.58 | | 1.32 |
|  | **Service worker** | | 1.25 | | 0.383 | 0.76 | | 2.07 |
|  | **Faculty member** | | 0.16 | | <0.001 | 0.07 | | 0.39 |
| **Workplace Distance from EHCSIR center** | | | 0.91 | | 0.037 | 0.82 | | 0.99 |
| **Workplace Social Capital** | | | 0.82 | | 0.508 | 0.45 | | 1.49 |
| ^a^ Random effect: type of center and center  ^b^ Random effect: center | | | | | | | | |
